# Influence of Network Synthesis Strategies on Liquid Crystal Elastomer Properties

**DOI:** 10.1021/acs.macromol.5c01037

**Published:** 2025-07-25

**Authors:** Rakine Mouhoubi, Jason Richard, Vincent Lapinte, Sébastien Blanquer

**Affiliations:** † Institut Charles Gerhardt Montpellier (ICGM), CNRS, Université de Montpellier, ENSCM, 34293 Montpellier, France; ‡ Institut Européen des Membranes (IEM), CNRS, Université de Montpellier, ENSCM, 34090 Montpellier, France

## Abstract

Network synthesis strategies are critical in determining the properties of liquid crystal elastomers (LCEs), making their selection essential for tailoring material performance. This study systematically compares chain extension and chain transfer approaches to identify key structural factors governing LCE behavior. By assessing how the chemical nature and length of liquid crystal oligomers in chain extension, as well as the proportion of pendant thiols in chain transfer, influence material properties, we establish cross-linking density, mesogen content, and mesogen distribution as key structural factors impacting LCE performance across both synthesis routes. To illustrate the practical significance of these findings, a LCE bilayer with shape-memory behavior is fabricated using two LCE networks with distinct thermomechanical properties, demonstrating controlled rolling deformation upon heating. Overall, this study provides a framework for designing LCEs with tailored properties by selecting the appropriate synthesis route.

## Graphical abstract

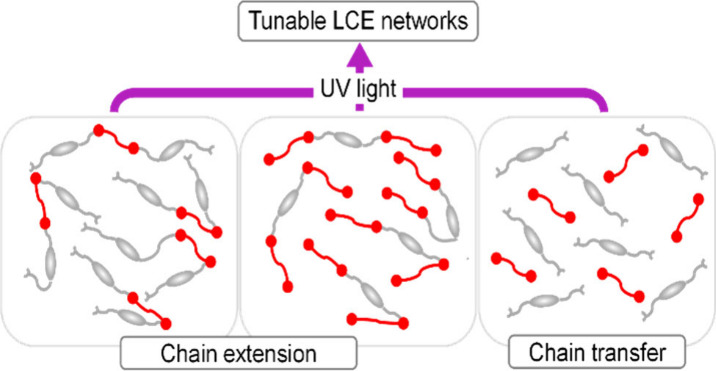

## Introduction

Liquid crystal elastomers (LCEs) are stimuli-responsive polymers that combine the anisotropic properties of liquid crystal molecules, known as mesogens, with the entropic elasticity of a lightly cross-linked polymer network. This combination enables LCEs to undergo large, rapid, and reversible deformations, resulting in both shape-memory and actuation behaviors. These effects result from the nematic-to-isotropic phase transition triggered by external stimuli such as heat or light, making LCEs promising candidates for applications in artificial muscles,^1^ sensors,^2^ and soft robotics.^3,4^ The nematic–isotropic transition temperature (*T*_NI_) defines the temperature at which this phase transition occurs. Below *T*_NI_, LCEs are in the nematic state, where mesogens interact through π–π stacking interactions. In monodomain nematic LCEs, mesogens maintain long-range orientational order, whereas in polydomain nematic LCEs, they are arranged in locally aligned domains without macroscopic order. Above *T*_NI_, these interactions are disrupted, and the mesogens adopt a random orientation in the isotropic state. This phase transition induces a pronounced conformational change in the polymer chains between cross-linking points, ultimately driving the macroscopic shape change.^5^

Therefore, the thermomechanical properties of LCEs are strongly influenced by how mesogens are incorporated into the polymer network, as the network structure governs mesogenic interactions and mobility. However, selecting a synthetic approach that is compatible with the desired alignment technique and also adaptable to 3D printing presents chemical constraints. In this context, the literature reports chemical strategies such as chain extension and chain transfer reactions that facilitate the fabrication of LCEs compatible with various alignment techniques, including mechanical,^6,7^ rheological,^8–11^ surface-enforced^12–14^ and magnetic field alignment.^15–17^ These approaches are also adaptable to 3D printing, particularly digital light processing, enhancing their processing versatility.^15,17–20^ Chain extension, based on the oligomerization of mesogens before cross-linking, offers a promising way to increase the molecular weight between cross-linking points in LCE networks.^5^ Two main strategies have been explored: aza-Michael and thiol-Michael addition reactions. In the first strategy, diacrylate mesogens undergo an aza-Michael addition with primary amines, followed by a secondary photopolymerization step to cross-link the acrylate-terminated oligomers.^21^ The thio-Michael addition reaction follows a similar concept, using mesogen oligomerization with a dithiol chain extender.^22^ This approach enables the synthesis of both thiol-terminated and acrylate-terminated liquid crystal oligomers (LCOs), providing greater control over network architecture. In parallel, chain transfer reactions provide an alternative route to LCE synthesis.^12^ Unlike chain extension, which requires an oligomerization step that can take several hours, chain transfer enables rapid network formation within seconds. This approach relies on the photo-cross-linking of mesogens with dithiols, influencing network formation by incorporating pendant thiol groups that impact the final material properties.

Despite some attempts to tailor the thermo-mechanical properties of main-chain LCEs prepared via classical approaches,^23–26^ comprehensive investigations are still lacking for chain extension and chain transfer synthesis routes. In the case of chain extension reactions, material properties are influenced by the chemical nature and length of LCOs.^22^ A recent study has compared aza-Michael and thio-Michael additions for the synthesis of acrylate-terminated LCOs, focusing on their effects on material properties and *stimulus* reponse.^27^ For chain transfer reactions, material properties are influenced by the proportion of pendant thiols. A recent study has investigated their influence on the glass transition temperature and elastic modulus.^12^ Overall, although chain extension and chain transfer are the most widely used methods for LCE synthesis across various alignment techniques, no comprehensive study has systematically evaluated the impact of these critical parameters on the thermal, mechanical, and thermo-mechanical properties of LCE networks. Moreover, all previous studies have only compared variations in network composition within a single synthesis route. This is because each synthesis method was historically associated with a specific alignment technique. However, recent advancements show that different chemistries are now being adapted to various alignment techniques. For instance, chain extension via thiol-Michael addition has recently been implemented in surface-enforced^28^ and magnetic alignment,^16^ while chain transfer has been introduced to magnetic field alignment through 3D printing.^15^ Consequently, the choice of synthesis route is no longer dictated solely by the constraints of the alignment technique but should also consider the targeted LCE properties, regardless of the alignment method used. Nevertheless, no study has systematically compared these two widely used LCE synthesis routes in terms of thermal, mechanical, and thermo-mechanical properties.

Herein, we investigate how different network synthesis strategies influence the properties of main-chain LCEs. Specifically, we compare LCEs prepared via chain extension and chain transfer to demonstrate how differences in network architecture impact the final material properties. By maintaining a consistent approach in monomer selection, solvent choice, and cross-linking method, this study isolates the key structural factors governing thermal, mechanical, and thermo-mechanical properties, independent of the alignment technique. To establish these key structural factors, we investigate the influence of critical parameters within each synthesis route on the properties of LCE networks. For chain extension, we examine the effect of the chemical nature and length of LCOs, while for chain transfer, we assess the impact of pendant thiol content and the occurrence of chain transfer events. As illustrated in Figure 1, these strategies result in different network architectures, leading to distinct material behaviors. Comparing and analyzing these differences enables the identification of the key structural factors common to both approaches. To highlight the practical relevance of these findings, we fabricate a LCE bilayer with shape-memory behavior using two distinct LCE networks. This bilayer exploits the contrasting thermo-mechanical responses of the two networks, enabling controlled rolling deformation during shape recovery, driven by their differing phase transition temperatures. Our study provides valuable guidelines for tailoring LCE properties simply by selecting the appropriate synthesis route.

**Figure 1:**
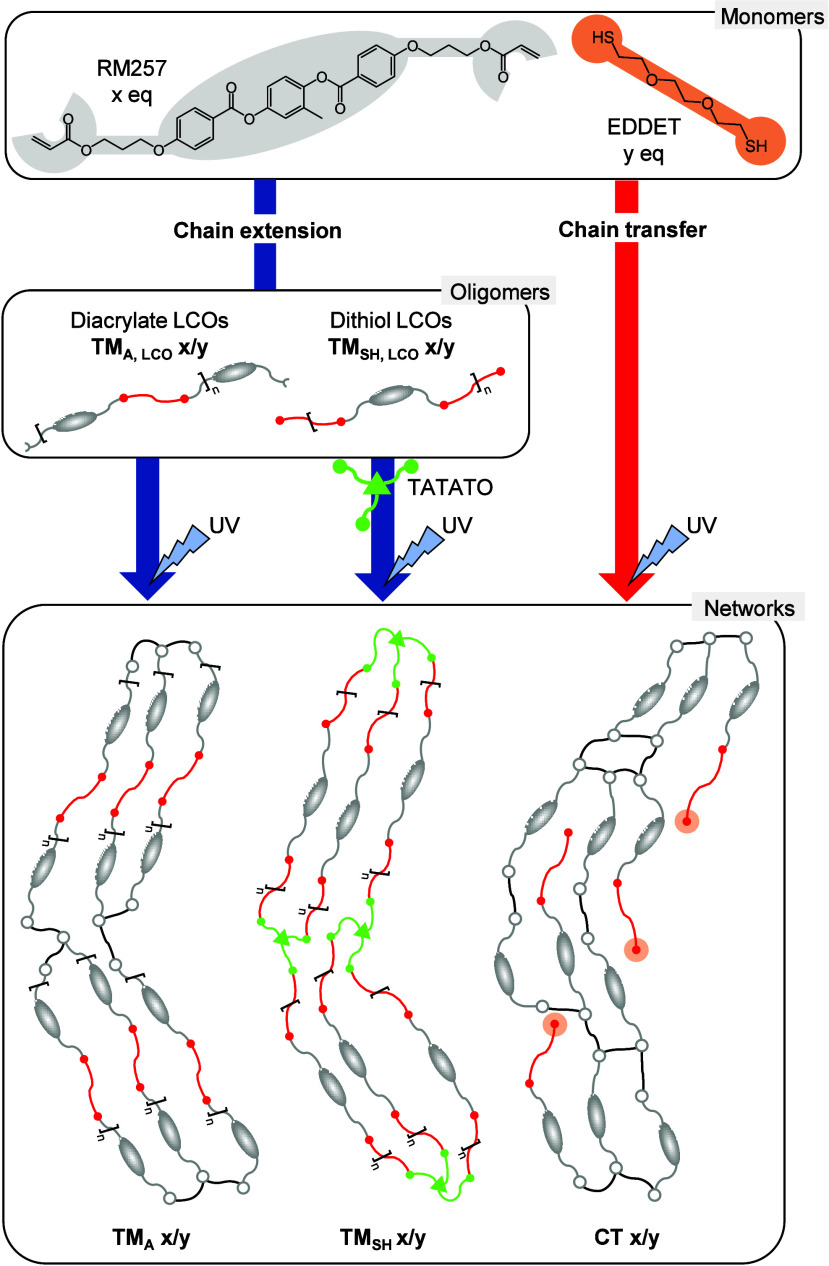
Schematic representation of the different approaches for obtaining LCE networks via chain extension and chain transfer. LCEs are labeled based on their synthesis route: TM_A_ x/y for those derived from diacrylate-terminated LCOs, TM_SH_ x/y for those from dithiol-terminated LCOs, and CT x/y for those obtained via chain transfer, where x/y represents the RM257/EDDET molar ratio.

## Experimental Section

### Chemicals

1,4-Bis­[4-(3-acryloyloxypropyloxy)­benzoyloxy]-2-methylbenzene (≥95%, RM257) was purchased from Smolecule (USA, San Antonio). 2,2-(Ethylenedioxy)­diethanethiol (≥95%, EDDET), 1,3,5-triallyl-1,3,5-triazine-2,4,6­(1*H*,3*H*,5H)-trione (≥98%, TATATO), 2,6-di-*tert*-butyl-4-methylphenol (≥99%, BHT), toluene (≥99.8%), and phenylbis­(2,4,6-trimethylbenzoyl)­phosphine oxide (≥97%, PPO) were purchased from Sigma-Aldrich (France). Triethylamine (≥99%, TEA) was purchased from Carlo Erba (France). All materials were used as received without further purification.

### Synthesis of LCOs

Diacrylate-terminated and dithiol-terminated liquid crystal oligomers (LCOs) with varying degree of polymerization (DP_n_) were synthesized via a base-catalyzed thio-Michael polyaddition by reacting RM257 with EDDET in different molar ratios based on Carother’s equation. For diacrylate-terminated LCOs, RM257 and EDDET were combined in molar ratios of 1.1/1, 1.25/1, and 1.5/1, while for dithiol-terminated LCOs, the ratios were 1/1.1, 1/1.2, and 1/1.4. In both cases, RM257 and toluene were mixed in a 1/1 wt % ratio in a 25 mL vial, followed by the addition of BHT (2 wt %) and PPO (2 wt %). The mixture was heated to 85 °C, vortex-mixed, and cooled to room temperature before adding EDDET. After further vortex mixing, TEA (9 mol %) was added dropwise to initiate polymerization. The reaction proceeded under continuous stirring at room temperature for 24 h. The resulting LCO inks were used without further purification. Diacrylate-terminated LCOs are labeled TM_A, LCO_ x/y, and dithiol-terminated LCOs are labeled TM_SH, LCO_ x/y, where x/y represents the RM257/EDDET molar ratio.

### Synthesis of LCEs by Chain Extension

To synthesize LCEs from diacrylate-terminated LCO inks, the inks were directly poured into a PTFE mold and photo-cross-linked under 365 nm UV light (20 mW/cm^2^) for 30 min using a UV cross-linker Bio-Link chamber (Thermo Fisher, France). For LCE synthesis from dithiol-terminated LCO inks, TATATO was first added as a cross-linker to ensure a balanced molar ratio between EDDET and the combined amount of RM257 and TATATO. The resulting mixture was then vortex-mixed, poured into a PTFE mold and photo-cross-linked for 2 h. The obtained samples were washed by swelling in toluene for 48 h to remove unreacted polymer residues and subsequently dried in a vacuum oven at 90 °C for 24 h. LCEs synthesized from diacrylate-terminated LCOs are labeled TM_A_ x/y, and LCEs synthesized from dithiol-terminated LCOs are labeled TM_SH_ x/y, where x/y represents the RM257:EDDET molar ratio.

### Synthesis of LCEs by Chain Transfer

LC inks were formulated by blending RM257 with EDDET in molar ratios of 1.1/1, 1/1.5, and 1/2. RM257 and toluene were first mixed in a 1/1 wt % ratio in a 25 mL vial, followed by the addition of BHT (2 wt %) and PPO (2 wt %). The mixture was heated to 85 °C for 5 min to reach the isotropic state, vortex-mixed, cooled to room temperature before EDDET was added, and further vortex-mixed. The LC inks were then poured directly into a PTFE mold and photo-cross-linked under 365 nm UV light. The obtained samples were washed by swelling in toluene for 48 h to remove unreacted polymer residues and subsequently dried in a vacuum oven at 90 °C for 24 h. LCEs synthesized from these LC inks are labeled CT x/y, where x/y represents the RM257/EDDET molar ratio.

### ^1^H NMR Characterization

^1^H NMR spectroscopy was employed to determine the degree of polymerization (DP_n_) and the number-average molecular weights (*M*_n_) of the LCOs through end-group analysis. The ^1^H NMR spectra were obtained using a 400 MHz Bruker’s Avance Spectrometer with CDCl_3_ as deuterated solvent. The chemical shifts of protons were relative to CHCl_3_ residual in CDCl_3_ at δ = 7.26 ppm. The DP_n_ value for each diacrylate-terminated LCO was calculated based on the integral ratio of the six protons in the diacrylate end-groups (protons b, c, and d in Figure 2b) to the four aromatic protons (protons a in Figure 2b) in the repeating mesogen unit, using eq 1:

1 $${\mathrm{DP}}_{n}=\frac{\frac{\int_{8.10}^{8.40}Ia}{4}}{\frac{\int_{5.87}^{5.98}Id+\int_{6.16}^{6.28}Ic+\int_{6.45}^{6.56}Ib}{6}}-1$$

**Figure 2:**
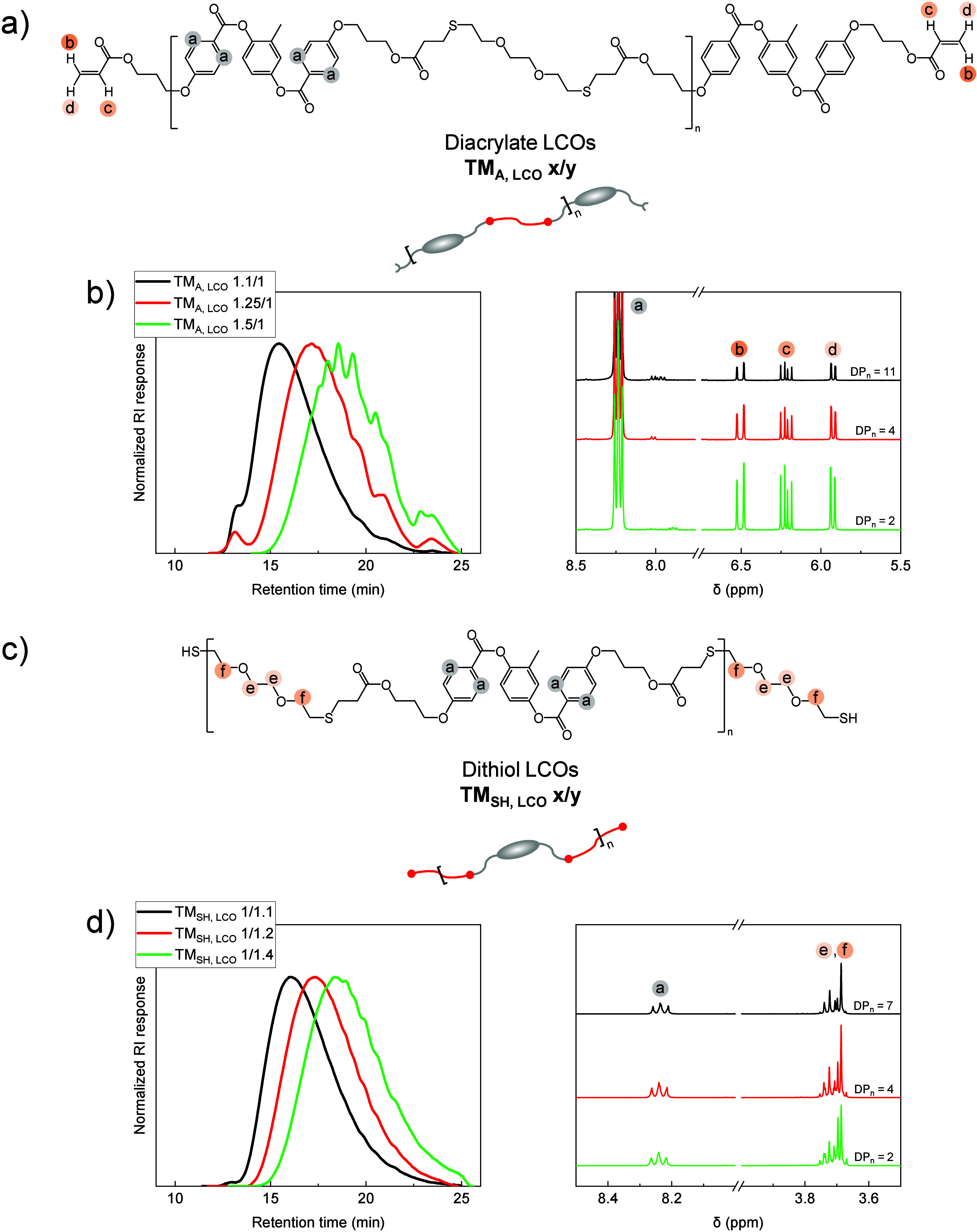
(a) Chemical structure of diacrylate-terminated LCOs, TM_A,LCO_ x/y, synthesized via base-catalyzed thiol-Michael polyaddition. (b) ^1^H NMR spectra and SEC traces of LCOs prepared with varying RM257/EDDET molar ratios. (c) Chemical structure of dithiol-terminated LCOs, TM_SH,LCO_ x/y, synthesized via base-catalyzed thiol-Michael polyaddition. (d) ^1^H NMR spectra and SEC traces of LCOs prepared with varying RM257/EDDET molar ratios.

The DP_*n*_ value for each dithiol-terminated LCO was calculated based on the integral ratio of the terminal methylene protons adjacent to the oxygen atoms of EDDET (protons e and f in Figure 2d) to the four aromatic protons (protons a in Figure 2d) in the repeating mesogen unit, using eq 2:

2 $${\mathrm{DP}}_{n}=\frac{\frac{\int_{8.10}^{8.40}Ia}{4}}{\frac{\int_{3.64}^{3.77}Ie,f-2\int_{8}^{8.40}Ia}{8}}$$

The *M*_n_ values for diacrylate-terminated LCOs and dithiol-terminated LCOs were calculated using eq 3:

3 $$M_{n}={\mathrm{DP}}_{n}\times M_{\mathrm{repeating unit}}+M_{\mathrm{end‐groups}}$$

where *M*_repeating unit_ and *M*_end‑groups_ are the molar masses of the repeating unit and the end groups, respectively. The molar mass of the repeating unit is 772 g/mol, while the molar masses of the end groups are 589 g/mol for diacrylate-terminated LCOs and 183 g/mol for dithiol-terminated LCOs.

### Size Exclusion Chromatography

Size exclusion chromatography (SEC) was used to characterize the molar mass distribution of the LCOs. The LCO inks were first precipitated in ethanol and the resulting LCOs were dried in an oven at 90 °C for 24 h. SEC measurements were performed on a Varian 390-LC apparatus with a refractive index detector from Agilent Technologies, equipped with two columns Infinitylab Oligopore. The eluent was DMF containing 0.1 wt % LiCl, with a flow rate of 0.8 mL/min at 40 °C. Samples were filtered using AIT France PTFE syringe filters (0.22 μm, 13 mm diameter) before the injection. Linear poly­(methyl methacrylate)­s (PMMA) were used as standards. The curves were normalized to maximum intensity.

### Gel Fraction and Swelling Ratio Tests

To determine gel fractions and swelling ratios, all LCE samples were dried in a vacuum oven at 90 °C for 24 h before the initial weight was determined (*m*_i_). The samples were then swelled in toluene for 48 h. They were then dried in a vacuum oven at 90 °C for 24 h before the dried weight was determined (*m*_d_). They were then swelled in toluene for 24 h and weighed in a swollen state (*m*_s_). The gel fraction and swelling ratio for each LCE sample was calculated using eqs 4 and 5:

4 $$\mathrm{gel fraction}=\frac{m_{d}}{m_{i}}\times 100$$

5 $$\mathrm{swelling ratio}=\frac{m_{s}-m_{d}}{m_{d}}\times 100$$

### Raman Spectroscopy

To assess the presence of dangling thiols in LCEs prepared by chain transfer, Raman Micro-Raman analyses were conducted using a HORIBA Jobin LabRAM HR800UV Raman spectrometer (λ = 660 nm, 300 gr mm^–1^ grating). Si vibration at 520 cm^–1^ was employed for calibration. For LC inks, the solutions were analyzed in a 1 cm cuvette. For LCEs, a Leica PL Fluotar × 50 objective (NA = 0.55) was used. Raman spectra were collected with an exposure time of 10 s and 9 accumulations at a laser power of 20 mW. Baseline correction was performed on the obtained data. The S–H vibration band *ν*_SH_ at 2577 cm^–1^ and the ester vibration band *ν*_ester_ at 1730 cm^–1^ were fitted with a pseudo-Voigt function, with the width of the function kept constant for all fits. The calculated intensities of the fits were then reported as *I*_SH_ and *I*_ester_ for the *ν*_SH_ and *ν*_ester_ bands, respectively. The conversion rate of thiol groups during photo-cross-linking was then calculated using eq 6:

6 conversion=(ISHIester)LCink−(ISHIester)LCE(ISHIester)LCE

where (ISHIester)LCink and (ISHIester)LCE are the intensity ratios between the S–H and the ester vibration bands for the LC ink and for the LCE, respectively.

### Photorheology

Photorheological measurements were conducted to investigate the photo-cross-linking behavior of LCE networks using a ThermoFisher HAAKE MARS 60 rheometer with a 20 mm parallel plate–plate geometry and a 0.5 mm gap. A UV mercury lamp (0.5 W/cm^2^) was used for irradiation. Data were recorded at a strain of 1% and a frequency of 1 Hz, ensuring measurements remained within the linear viscoelastic region, which was determined beforehand through a strain amplitude sweep. An equilibration time of 50 s was maintained before switching on the UV lamp. The photo-cross-linking rate (*R*_*G*′_) was determined as the maximum value of d*G*′/d*t*, while the final storage modulus (*G*′_final_) was taken as the last plateau value.

### Differential Scanning Calorimetry

Differential scanning calorimetry (DSC) was used to characterize the thermal transitions and properties of the LCOs and LCEs. DSC analyses were carried out using a Netzsch DSC 3500 Sirius. The ethanol-precipitated and dried LCOs or LCEs with a mass between 5 and 10 mg were loaded into standard aluminum DSC pans. The samples were equilibrated at −50 °C and heated to 120 °C at a rate of 10 °C/min. They were then cooled slowly to −50 °C at a rate of 2 °C/min to allow LC self-assembly. They were then heated to 120 °C at a rate of 20 °C/min. Data were reported from the second heating scans. The glass transition temperature (*T*_g,DSC_) was defined at the step change in the slope of the heat flow signal. The nematic–isotropic transition temperature (*T*_NI,DSC_) was determined at the minimum value of the endothermic peak. The enthalpy change (Δ*H*_NI_) was measured by integrating the endothermic energy well of the nematic–isotropic transition.

### Uniaxial Tensile Testing

Uniaxial tensile tests were performed to study the mechanical properties of LCEs. The tensile tests were executed on an Instron 3366L5885 mechanical tester equipped with a 100 N load cell. Rectangular samples measuring approximately 20 × 1 x 1 mm^3^ were tested at a displacement rate of 5 mm/s. The Young modulus was measured by taking the slope in the linear region of the stress–strain curve. The threshold stress was determined as the stress at the intersection of the tangents of the first linear region and the soft elasticity plateau. The failure strain value was defined as the maximum strain at break.

### Dynamic Mechanical Analysis

Dynamic mechanical analysis (DMA) was employed to study the thermo-mechanical properties and the phase transitions of the LCEs. DMA tests were performed using a Mettler Toledo DMA instrument in tensile mode. Rectangular samples measuring approximately 20 × 1 × 1 mm^3^ were tested with an active length of 10 mm. The samples were equilibrated at −50 °C. They were then subjected to a strain of 0.2% at 1 Hz and heated from −50 to 120 °C at a rate of 2 °C/min. *T*_g,DMA_ was defined at the maximum value of the loss tangent (tan δ) curve. *T*_NI,DMA_ was attributed to the lowest value of the storage modulus (*E*′) curve above *T*_g,DMA_. The nematic modulus (*E*′_25 °C_) was measured using the storage modulus value at 25 °C. The dissipation factor, defined as the area under the loss tangent curve, was calculated for each network using a baseline correction from −40 °C to *T*_NI,DMA_ + 20 °C.

### Fixity and Shape Recovery Tests

Fixity measurements were conducted to evaluate the shape retention of LCE samples in the nematic state, specifically TM_A_ 1.1/1, TM_A_ 1.25/1, TM_SH_ 1/1.1, TM_SH_ 1/1.2, CT 1.1/1, and CT 1/1.5. DMA tests were performed using a TA Instruments DMA Q850 in tensile mode. Rectangular samples measuring approximately 20 × 1 × 1 mm^3^ were tested with an active length of ∼7 mm. Each sample was first equilibrated at *T*_NI,DMA_ + 10 °C, then strained at a rate of 10%/min until reaching 100% engineering strain. The stress was then immediately released to 0 N and the samples were allowed to recover freely in a relaxed state for 1 h, until the engineering strain stabilized. The fixity value (*R*_f_) was determined as the final strain plateau value after 1 h of stabilization. Shape recovery measurements were performed for TM_A_ 1.1/1 and TM_SH_ 1/1.2 to study the thermal recovery behavior of both networks, with the aim of fabricating an LCE bilayer actuator. After the fixity procedure was completed, the strained samples, in a stress-free state, were heated at a rate of 10 °C/min until they reached their maximum possible recovery.

## Results and Discussion

In this work, we focused exclusively on main-chain LCEs. Various strategies have been reported for integrating mesogens into polymer networks, including end-on, side-on, and main-chain attachments.^29^ Among these architectures, main-chain LCEs, where mesogens are directly incorporated into the polymer backbone, have been the most widely studied due to their strong through-bond coupling. This architecture is particularly advantageous for promoting mesogen alignment within network, thereby improving actuation performance.^30^ We also limited our comparison to two commonly used synthetic approaches: chain extension and chain transfer reactions. Historically, main-chain LCEs have been synthesized via hydrosilylation reactions,^31^ or more recently by a two-stage thiol–acrylate Michael addition and photopolymerization (TAMAP) approach.^32^ However, both methods rely on mechanical alignment during the second polymerization step, limiting their scalability and compatibility with other alignment techniques, such as surface-enforced, rheological, magnetic, or electric field alignment, and makes them difficult to adapt for 3D printing.

To ensure a meaningful comparison between LCE networks obtained via chain extension and chain transfer, we maintained a consistent approach in the selection of materials and experimental conditions. All the networks were polydomain LCEs synthesized in the isotropic state, where the presence of solvent disrupted LC order during network formation. It is important to specify this point, as the synthesis conditions, whether in the nematic or isotropic state, can influence the final properties of LCE networks.^23^ To guarantee uniform curing conditions, photo-cross-linking was used for all networks. RM257, a widely used mesogen, and EDDET, a common chain extender, were selected as monomers based on their prevalence in the literature.^1,8,18–20,23,25,32–39^ Toluene was used as the solvent, and the initial dilution of the monomers was kept the same for all networks to eliminate variability due to concentration effects. Whether formed via chain extension or chain transfer, all LCE networks were obtained by photo-cross-linking their precursors under 365 nm UV light. LCEs are labeled based on their synthesis route: TM_A_ x/y for those derived from diacrylate-terminated LCOs, TM_SH_ x/y for those from dithiol-terminated LCOs, and CT x/y for those obtained via chain transfer, where x/y represents the RM257/EDDET molar ratio.

### Main-Chain LCEs via Chain Extension

While both strategies lead to the formation of LCEs, chain extension and chain transfer differ fundamentally in their synthetic approach, which impacts the structure and properties of the resulting networks. Unlike LCEs prepared via chain transfer, those obtained via chain extension require an additional precursor synthesis step before photo-cross-linking, as shown in Figure 1. These precursors are LCOs, which play a critical role in defining the properties of LCEs produced through this method. Indeed, in LCEs formed by step-growth polymerization, both the DP_*n*_ and the chemical nature of LCOs are critical parameters in determining the final material properties. In particular, end-groups strongly influence LCO behavior and, consequently, the resulting LCE properties. Therefore, to explore the full range of LCEs that can be synthesized via chain extension, diacrylate-terminated LCOs and dithiol-terminated LCOs with varying DP_*n*_ were prepared via base-catalyzed thio-Michael polyaddition, reacting RM257 with EDDET in different molar ratios. Diacrylate-terminated LCOs were obtained with a molar excess of RM257 over EDDET, while dithiol-terminated LCOs were produced with a molar excess of EDDET over RM257. Specifically, diacrylate-terminated LCOs were synthesized using RM257/EDDET molar ratios of 1.1/1, 1.25/1, and 1.5/1, and are labeled TM_A, LCO_ x/y, where x/y represents the RM257/EDDET molar ratio. Similarly, dithiol-terminated LCOs were obtained with RM257/EDDET molar ratios of 1/1.1, 1/1.2, and 1/1.4, and are labeled TM_SH, LCO_ x/y, where x/y represents the RM257/EDDET molar ratio. These molar ratios were selected within the limits of chain extension. When the composition approaches stoichiometric balance, the LCO solution becomes highly viscous, hindering the photo-cross-linking due to reduced precursor mobility. Conversely, deviating too far from the stoichiometric ratio results in a cross-linked material that is overly rigid and lacks flexibility. The obtained LCOs were then characterized by ^1^H NMR spectroscopy to determine the DP_*n*_ (with one repeating unit illustrated in Figure 2a for TM_A,LCO_ and in Figure 2c for TM_SH,LCO_) and the *M*_n_ by end-group detection, as detailed in the Experimental Section. The values are summarized in Table 1, and the full ^1^H NMR spectra are provided in Figure S1 for TM_A,LCO_ and in Figure S2 for TM_SH,LCO_. For TM_A,LCO_, DP_*n*_ values were determined based on the integral ratio of the six protons in the diacrylate end-groups (protons b, c, and d) to the four aromatic protons (protons a) in the repeating mesogen unit, as shown in Figure 2b. For TM_SH,LCO_, DP_*n*_ values were calculated from the integral ratio of the terminal methylene protons adjacent to the oxygen atoms of EDDET (protons e and f) to the four aromatic protons (protons a) in the repeating mesogen unit, as shown in Figure 2d. For both TM_A,LCO_ and TM_SH,LCO_, DP_*n*_ decreases as the molar ratio deviates further from stoichiometry by adding an excess of either RM257 or EDDET. This trend observed in ^1^H NMR spectroscopy was further validated by SEC analysis, as shown in Figure 2b for TM_A,LCO_ and in Figure 2d for TM_SH,LCO_. The broad molecular weight distributions in the SEC traces reflect the characteristics of step-growth polymerization. Moreover, as summarized in Table 1, lower DP_*n*_ values correlate with a narrower molecular weight distribution, whereas higher DP_*n*_ results in an increase the dispersity index (*Đ*) as the system approaches stoichiometric balance. Additionally, for LCOs with small DP_*n*_, the SEC traces show distinct peaks corresponding to individual oligomeric species, where each peak represents a specific DP_*n*_ value, reflecting the separation capability of the column.

**Table 1:** Summary of ^1^H NMR^a^ and DSC Data for TM_A,LCO_ x/y and TM_SH,LCO_ x/y Characterization

	DP_*n*_ ^a^ calcd	*M*_n_ calcd^b^ (g/mol)	*Đ* ^c^	*T*_g,DSC_ ^d^ (°C)	*T*_NI,DSC_ ^d^ (°C)	Δ*H*_NI_ ^d^ (J/g)
TM_A,LCO_ 1.1/1	11	9000	7.4	–8	66	1.32
TM_A,LCO_ 1.25/1	4	3700	3.6	–11	75	1.44
TM_A,LCO_ 1.5/1	2	2100	1.6	–11	80	1.79
TM_SH,LCO_ 1/1.1	7	5600	4.7	–12	38	0.94
TM_SH,LCO_ 1/1.2	4	3300	3.8	–16	11	0.42
TM_SH,LCO_ 1/1.4	2	1700	3.6	–21	–2	0.12

a Calculated according to eqs 1 and 2 from ^1^H NMR spectra.

b Calculated according to eq 3 from ^1^H NMR spectra.

c Determined by SEC.

d Determined by DSC.

For LCEs prepared *via* chain extension from TM_A, LCO_, photo-cross-linking occurred through the homopolymerization of excess acrylates, leading to high conversion, as indicated by gel fractions exceeding 93% (Table 2). This confirms the efficient cross-linking of TM_A_ networks. For LCEs prepared *via* chain extension from TM_SH, LCO_, a triallyl cross-linker (TATATO) was required to cross-link the excess thiols. To ensure complete thiol conversion, TATATO was added to balance the molar ratio between EDDET and the total amount of RM257 and TATATO. In this case, photo-cross-linking follows a thiol–ene mechanism. TM_SH_ networks exhibited gel fractions above 89%, confirming efficient cross-linking (Table 2).

**Table 2:** Summary of Gel Fraction and Swelling Ratio Tests, Photorheological Measurements and DSC Data for LCE Characterization

	gel fraction (%)	swelling ratio (%)	*R*_*G*′_ ^a^ (kPa/s)	*G*′_final_ ^a^ (MPa)	*T*_g,DSC_ ^b^ (°C)	*T*_NI,DSC_ ^b^ (°C)	Δ*H*_NI_ ^c^ (J/g)
TM_A_ 1.1/1	93.4 ± 0.1	89 ± 2	4	3.4 × 10^–1^	–3	73	2.16
TM_A_ 1.25/1	95.2 ± 0.2	70 ± 4	10	1.0	1	77	1.29
TM_A_ 1.5/1	95.1 ± 0.3	39 ± 4	22	1.7	13	95	0.41
TM_SH_ 1/1.1	89.6 ± 0.3	100 ± 4	0.02	5.2 × 10^–2^	–5	43	0.80
TM_SH_ 1/1.2	92.5 ± 0.3	90 ± 1	0.04	1.7 × 10^–1^	–5	31	0.49
TM_SH_ 1/1.4	94.3 ± 0.4	84 ± 2	0.12	4.4 × 10^–1^	–1	27	0.47
CT 1.1/1	92.7 ± 0.7	57 ± 2	61	1.1	9	114	0.20
CT 1/1.5	94.4 ± 0.9	61 ± 2	20	3.2 × 10^–1^	–8	59	0.21
CT 1/2	95.4 ± 0.8	86 ± 2	2	3.3 × 10^–2^	–16	38	0.95

a Calculated according to eqs 4 and 5.

b Determined by photorheological measurements.

c Determined by DSC.

### Main-Chain LCEs via Chain Transfer

For LCEs prepared *via* chain transfer, photo-cross-linking occurred directly from the initial monomer mixture, a LC ink composed of RM257 and EDDET. In general, with thiol–acrylate photopolymerization, a photoinitiated radical first generates a thiyl radical, which then reacts with an acrylate, leading to the formation of a carbon-centered radical. At this stage, propagation through acrylate homopolymerization can occur when the carbon-centered radical reacts with an unreacted acrylate. Simultaneously, the carbon-centered radical can undergo chain transfer to another thiol, regenerating the thiyl radical and sustaining the polymerization process.^40^ When using EDDET, both thiol end-groups are expected to participate in the photopolymerization process. However, a previous study by Hebner et al. has shown that in the case of LC monomers, dithiols tend to react only once, as their mobility is restricted by the rigidity of the mesogen.^12^ Consequently, LCEs obtained *via* chain transfer contain dangling thiol bonds, which play a critical role in defining LCE properties. The photo-cross-linking mechanism of RM257 and EDDET in our system is illustrated in Figure 3a. To explore the full range of LCEs that can be synthesized *via* chain transfer, LC inks were formulated by blending RM257 with EDDET with RM257/EDDET molar ratios of 1.1/1, 1/1.5, and 1/2. These molar ratios were selected within the limits of chain transfer. A higher excess of EDDET prevents photo-cross-linking, while a greater excess of RM257 results in a highly cross-linked material, which becomes too brittle. The presence of dangling thiols in LCE networks prepared *via* chain transfer was confirmed by Raman spectroscopy. Measurements were conducted on LC inks and their corresponding photo-cross-linked CT networks. The full Raman spectra of LC inks and CT networks are provided in Figure S3. As shown in Figure 3b, the S–H peak at 2577 cm^–1^, corresponding to the stretching vibration of the S–H bond, is clearly present in the fully cross-linked materials, indicating incomplete thiol conversions. This is further supported by Figure 3c, which illustrates the evolution of the *I*_*SH*_/*I*_*ester*_ intensity ratio, where the S–H peak is normalized to the ester peak at 1730 cm^–1^. In LC inks, the *I*_*SH*_/*I*_*ester*_ intensity ratio is directly proportional to the EDDET/RM257 ratio and is thus a good indicator of the relative amount of thiol functions in the sample (Figure S3c). A clear trend in thiol incorporation is observed as the amount of EDDET increases relative to RM257, with the *I*_SH_/*I*_ester_ ratio gradually increasing in both LC inks and CT networks. As a result, higher initial thiol content in LC inks leads to higher free thiols amounts in CT networks. However, thiol conversion does not appear to be dependent on the RM257/EDDET molar ratio, with conversion values between 45 and 65%, as calculated from eq 6. Despite the presence of unreacted thiols, efficient cross-linking was achieved, as evidenced by gel fractions values which approach 100% for all CT networks (Table 2), indicating nearly complete dithiol incorporation. This confirms that EDDET reacts only once, likely due to restricted mobility caused by the rigidity of RM257, leaving the second thiol end unreacted as a dangling thiol within the CT network.

**Figure 3:**
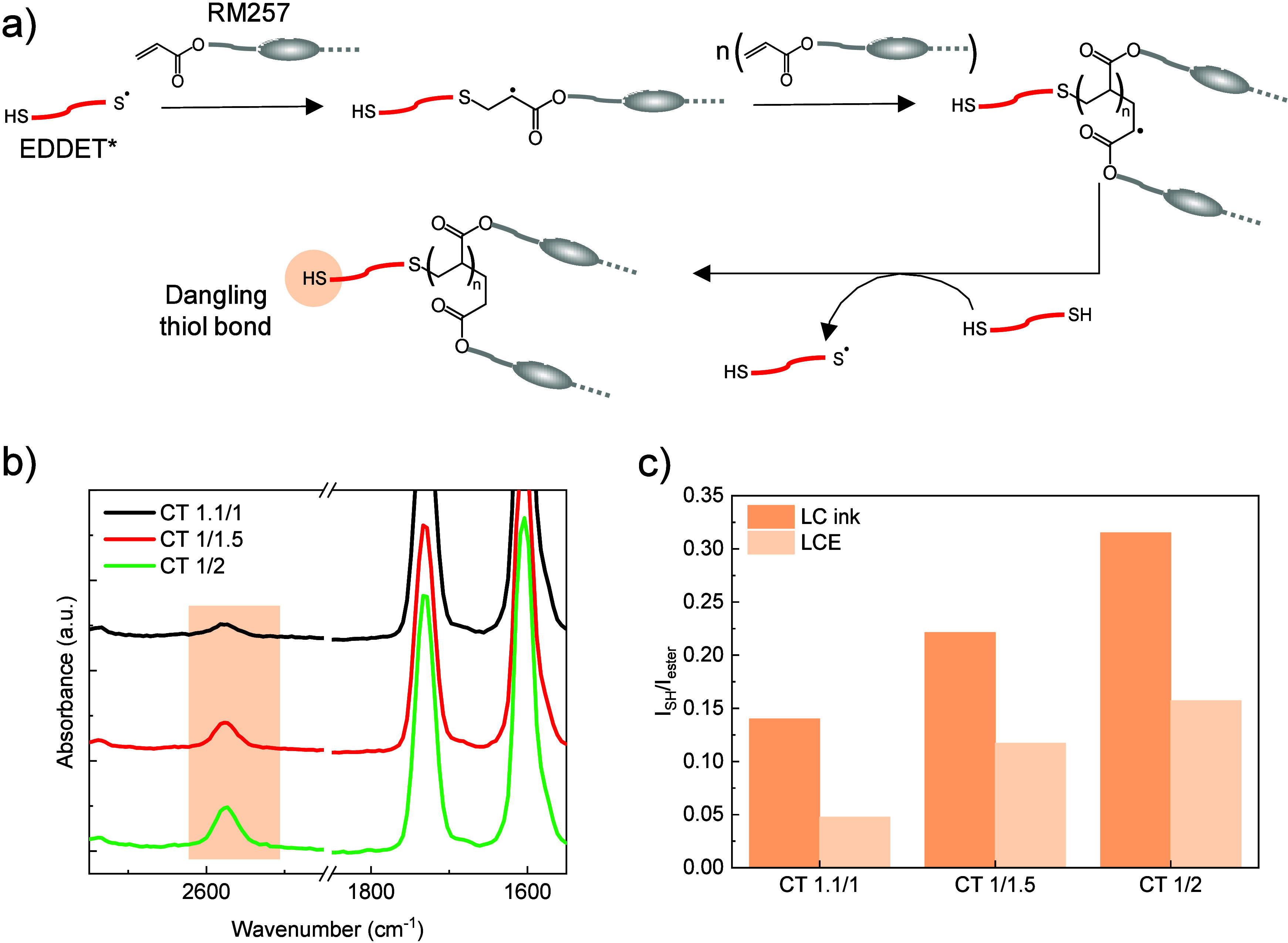
(a) Schematic representation of the photo-cross-linking mechanism of RM257 and EDDET for the fabrication of CT networks. (b) Raman spectra of CT networks. (c) Evolution of the *I*_SH_/*I*_ester_ intensity ratio as a function of the RM257/EDDET ratio for LC inks and CT networks.

### Cross-Linking Behavior of LCEs in Relation to the Chemical Strategy

Since the networks are built through different photopolymerization mechanisms, the photopolymerization rate of these LCEs was then evaluated using photorheology experiments. The photorheology curves of all networks are presented in Figure 4a. The values of the photo-cross-linking rate (*R*_*G*′_), defining the photopolymerization rate calculated as the maximum value of d*G*′/d*t*, and the final storage modulus (*G*′_final_), are summarized in Table 2 for all networks. For LCEs prepared *via* chain extension, the networks form more rapidly when their LCO precursors are shorter, as indicated by higher *R*_*G*′_ values. This is attributed to the higher mobility of smaller LCOs, which facilitates photopolymerization. For LCEs prepared *via* chain transfer, an increase in EDDET content leads to a decrease in *R*_*G*′_. Since this cross-linking occurs through acrylate homopolymerization, a higher EDDET content reduces the number of acrylate groups available for reaction, slowing down the formation of a cross-linked network. Overall, the formation of CT and TM_A_ networks results in the highest *R*_*G*′_ values, with higher *R*_*G*′_ values favored by the photo-cross-linking of smaller mesogenic units and increased mesogen content. Regardless of composition, all CT networks reach a plateau in *G*′ values in just 30 s, suggesting that cross-linking is completed within this short time frame. The formation of TM_A_ networks through acrylate homopolymerization is also relatively fast, with *R*_*G*′_ values comparable to those of CT networks. However, the plateau in *G*′ values is reached later, from 150 s for TM_A_ 1.5/1 up to 170 s for TM_A_ 1.1/1. Finally, the formation of TM_SH_ networks exhibits the slowest photopolymerization rates, with *R*_*G*′_ values 2 orders of magnitude lower than those of the other networks. Moreover, the plateau in *G*′ values is barely reached, even after two hours. This suggests that photo-cross-linking takes much longer in TM_SH_ networks, making high conversion more difficult to achieve, even though the resulting LCE networks exhibit high gel fractions. Since all networks were synthesized from the same initial dilution of EDDET and RM257, the *G*′_final_ values provide insight into the cross-linking density for each synthesis route (Table 2). For LCEs prepared *via* chain extension, *G*′_final_ for TM_A_ and TM_SH_ networks are higher when LCO precursors are shorter. This is attributed to the fact that targeting shorter oligomers results in a higher number of oligomer chains, leading to a higher concentration of reactive acrylate or thiol terminal groups per unit volume. As a result, the higher availability of reactive groups provides more cross-linking points, leading to a denser network. For LCEs prepared *via* chain transfer, *G*′_final_ for CT networks decrease as the amount of EDDET increases. This is because a higher EDDET content results in a higher number of chain transfer events, which reduces the number of cross-linking points, leading to a less dense network. Swelling tests confirmed the trends observed in photorheology regarding the cross-linking density of the networks (Table 2). TM_A_ 1.1/1, TM_SH_ 1/1.1, and CT 1/2 exhibit the highest swelling ratios in their respective synthesis routes, indicating the lowest cross-linking densities. Conversely, TM_A_ 1.5/1, TM_SH_ 1/1.4, and CT 1.1/1 show the lowest swelling ratios, indicating the highest cross-linking densities. Among the different synthesis routes, TM_SH_ networks appear to have the lowest cross-linking density. The modest differences in swelling ratios, all below 100%, can be explained by the low *M*_n_ values of the LCOs, which result in a relatively high density of cross-linking points.

**Figure 4:**
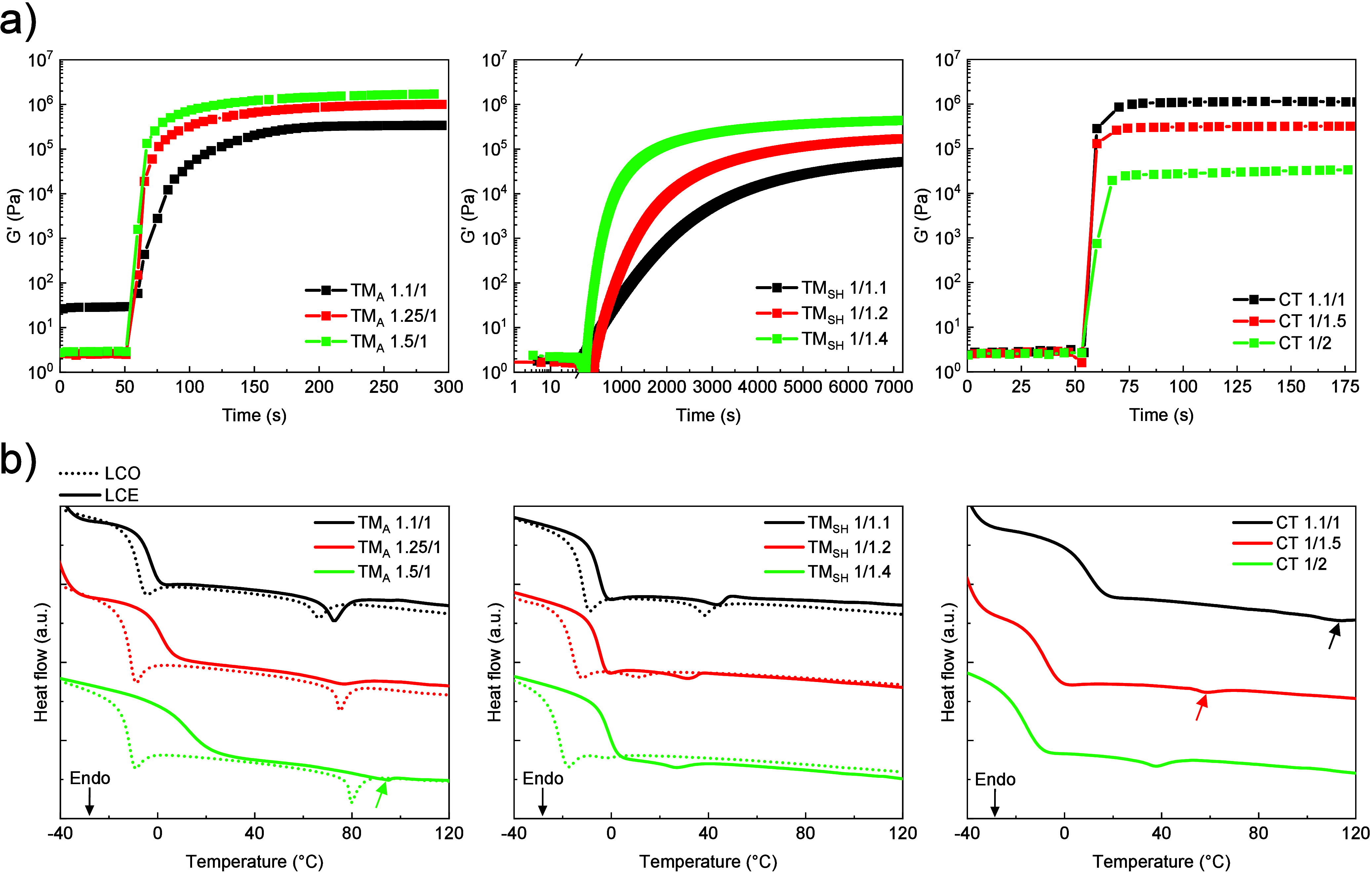
(a) Photorheological curves showing the evolution of *G*′ as a function of time during the formation of different LCE networks, with the UV lamp switched on after 50 s. (b) DSC traces of LCO precursors and LCE networks. Colored arrows indicate *T*_NI,DSC_ where it is not easily observable.

### Thermal Properties of the Synthesized LCEs

Since the phase transition behavior of the networks is expected to be influenced by both the photopolymerization mechanism and the phase transitions of their precursors, the thermal properties of both the precursors and the LCEs were analyzed by DSC (Figure 4b). The values of *T*_g,DSC_, *T*_NI,DSC_, and Δ*H*_NI_ are listed in Table 1 for TM_A,LCO_ and in Table 2 for TM_A_, TM_SH_ and CT networks.

For LCEs synthesized *via* chain extension, differences in phase transitions for TM_A,LCO_ and TM_SH,LCO_ precursors can be first assessed. For TM_A,LCO_, *T*_g,DSC_ varies little, decreasing slightly from −8 to −11 °C as DP_*n*_ decreases from 11 to 2, whereas for TM_SH,LCO_, *T*_g,DSC_ decreases more clearly from −12 to −21 °C as DP_*n*_ decreases from 7 to 2. In both cases, the decrease in *T*_g,DSC_ with decreasing DP_*n*_ is attributed to the higher molecular mobility of shorter LCOs. The lower *T*_g_ values and the more pronounced variation in *T*_g_ values observed for TM_SH,LCO_ can be explained by the absence of mesogens at the end-groups, which reduces molecular organization and increases chain flexibility. Regarding the nematic-to-isotropic transition, TM_A,LCO_ exhibit an increase in *T*_NI,DSC_ from 66 to 80 °C, with the associated Δ*H*_NI_ also increasing as DP_*n*_ decreases. This is because targeting shorter TM_A, LCO_ results in a higher molar content of RM257. The increased mesogen content enhances π–π stacking interactions, as evidenced by the higher enthalpy changes, resulting in a higher *T*_NI,DSC_. Conversely, TM_SH,LCO_ show a decrease in *T*_NI,DSC_ from 38 to −2 °C, with a corresponding reduction in Δ*H*_NI_ as DP_*n*_ decreases. TM_SH,LCO_ exhibit markedly lower *T*_NI,DSC_ values compared to TM_A,LCO_, as each oligomer contains one less mesogenic unit for the same chain length. Moreover, targeting shorter TM_SH,LCO_ results in a lower RM257 molar content. The reduced mesogen content weakens π–π stacking interactions, as evidenced by the lower enthalpy changes, resulting in a lower *T*_NI,DSC_.

Regarding LCE networks, differences in phase transition behavior can also be evaluated. For TM_A_ networks, *T*_g,DSC_ increases markedly from −3 to 13 °C as the DP_*n*_ of TM_A,LCO_ precursors decreases from 11 to 2, whereas for TM_SH_ networks, *T*_g,DSC_ increases only slightly from −5 to −1 °C as the DP_*n*_ of TM_SH,LCO_ precursors decreases from 7 to 2. In both cases, the increase in *T*_g,DSC_ with decreasing DP_*n*_ is attributed to the fact that targeting shorter oligomers results in a higher cross-linking density, as previously stated. This strongly restricts chain mobility, resulting in a higher *T*_g,DSC_. The higher *T*_g,DSC_ values and more pronounced variation in *T*_g,DSC_ values observed for TM_A_ networks are attributed to the fact they have a higher cross-linking density than TM_SH_ networks. For CT networks, *T*_g,DSC_ decreases drastically from 9 to −16 °C as the thiol content in the LC ink increases. This is attributed to the fact that higher thiol content leads to the consumption of more cross-linking points, since more chain transfer events happen and interfere with cross-linking. As a result, a less dense cross-linked network is formed, resulting in a lower *T*_g,DSC_. Additionally, the presence of pendant thiols increases free volume within the network, which also contributes to the drastic decrease in *T*_g,DSC_.

Regarding the nematic-to-isotropic transition, it is first observed that an increase in cross-linking density leads to a lower Δ*H*_NI_, regardless of the synthesis route. While this trend was previously observed,^25^ its explanation remained limited as it was studied within a single synthesis route. Comparing multiple strategies offers a more complete understanding. While Δ*H*_NI_ is related to mesogen content, as observed for LCOs, since it represents the energy required to disrupt local mesogen ordering, this trend suggests that cross-linking density also strongly influences the energy absorbed by the mesogens during the nematic-to-isotropic transition. In LCEs with lower cross-linking density, such as TM_A_ 1.1/1, TM_SH_ 1/1.1, and CT 1/2, mesogens are less constrained by the network, allowing them to easily undergo the nematic-to-isotropic transition. This results in a higher Δ*H*_NI_. Conversely, in LCEs with higher cross-linking density, such as TM_A_ 1.5/1, TM_SH_ 1/1.4, and CT 1.1/1, the restricted mobility imposed by the network prevents mesogens from undergoing the nematic-to-isotropic transition to the same extent. This results in a lower Δ*H*_NI_.

Then, more specifically for LCEs synthesized *via* chain extension, *T*_NI,DSC_ values are higher for the networks than for their LCO precursors, as cross-linking restricts chain mobility and promotes mesogenic interactions. However, they follow the same trends in *T*_NI,DSC_ values as their corresponding LCO precursors. TM_A_ networks exhibit an increase in *T*_NI,DSC_ from 73 to 95 °C as DP_*n*_ of TM_A,LCO_ decreases. In this case, a lower DP_*n*_ corresponds to a higher RM257 molar content. Conversely, TM_SH_ networks show a decrease in *T*_NI,DSC_ from 43 to 27 °C as DP_*n*_ of TM_SH,LCO_ decreases. In this case, a lower DP_*n*_ results in a lower RM257 molar content. For CT networks, *T*_NI,DSC_ decreases from 114 to 38 °C as the RM257 molar content is progressively reduced. These results show that LC content governs *T*_NI_ in these networks. As the mesogenic unit proportion in the network increases, mesogenic interactions become stronger. As a result, more thermal energy is required to disrupt these interactions, leading to a higher *T*_NI_. Additionally, while TM_A_ networks exhibit relatively high *T*_NI_ values and TM_SH_ networks show particularly low *T*_NI_ values, CT networks enable the widest range of *T*_NI_ values simply by varying the EDDET molar content. However, LC content is not the only factor governing *T*_NI_. Although CT 1/2 has the lowest mesogen content, its *T*_NI_ remains higher than that of TM_SH_ 1/1.2 and TM_SH_ 1/1.4. This is because, in CT networks, unlike those synthesized *via* chain extension, all mesogens are directly attached to the network, since their terminal ends act as potential cross-linking points during acrylate homopolymerization. Therefore, the cross-linking points enhance mesogenic interactions, maintaining a higher *T*_NI_.

Overall, the comparison between synthesis routes regarding the thermal properties of LCE networks allowed the identification of several key structural factors. Cross-linking density is a key structural factor governing *T*_g_ and Δ*H*_NI_. As cross-linking density decreases, LCEs exhibit lower *T*_g_ and higher Δ*H*_NI_. The proportion of mesogenic units within the network is a key structural factor governing *T*_NI_. As the proportion of mesogenic units within the LCE network increases, *T*_NI_ increases. The distribution of mesogens within the network is a key structural factor also governing *T*_NI_. For the same LC content, a LCE network with more cross-linking points derived from mesogens will exhibit a higher *T*_NI_ because of stronger mesogenic interactions.

### Mechanical and Thermomechanical Properties of the Synthesized LCEs

To investigate the influence of the synthesis route on the mechanical properties of LCEs under static loading conditions, tensile tests were performed (Figure 5a). The values of Young modulus, threshold stress (i.e., the stress at which the soft elasticity region, characterized by a nearly constant stress, begins), and failure strain are listed in Table 3.

**Figure 5:**
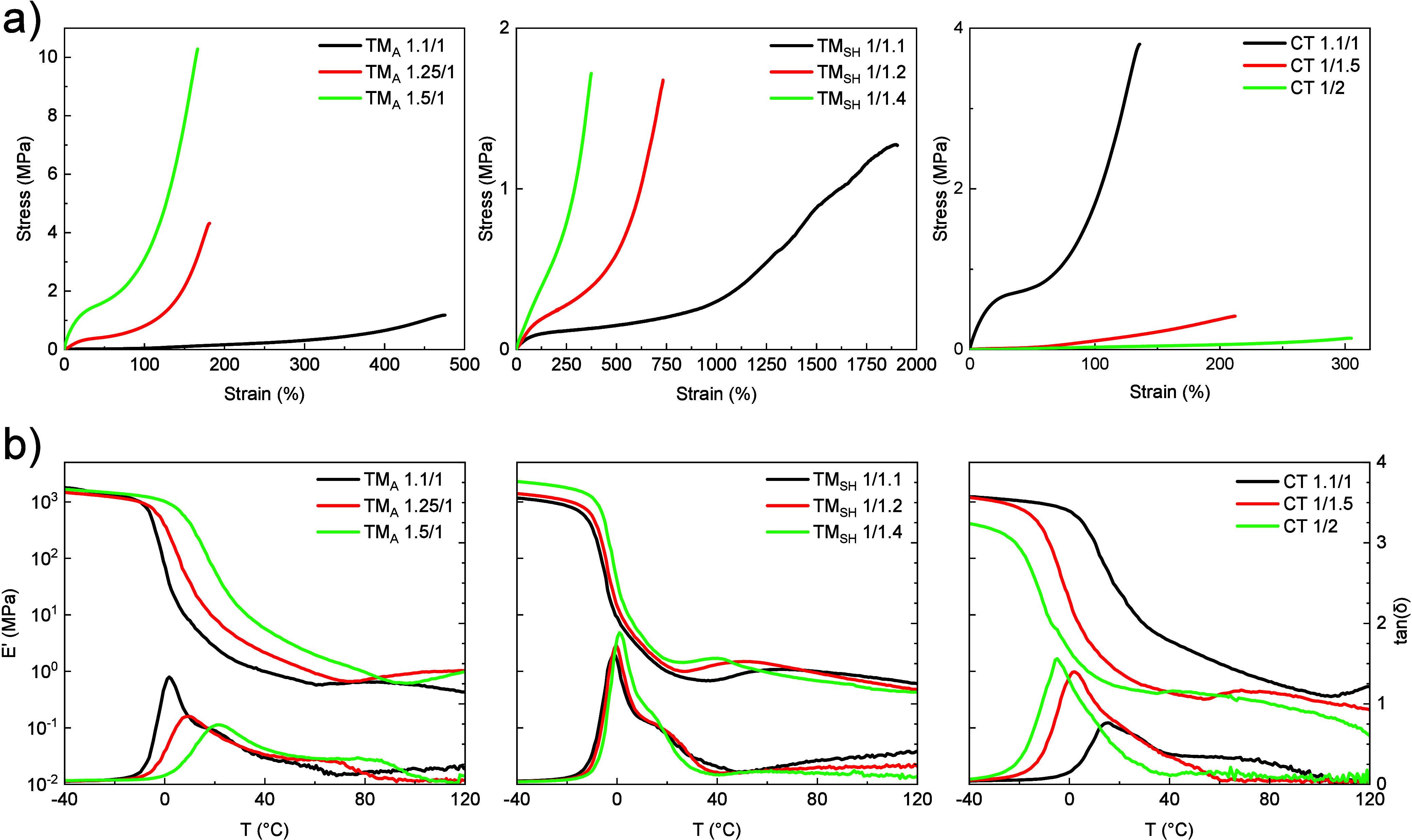
(a) Stress–strain curves of LCE networks. (b) Storage modulus (*E*′) and loss tangent (tan δ) traces of LCE networks.

**Table 3:** Summary of Tensile Test and DMA Data for LCE Characterization

	Young modulus^a^ (MPa)	threshold stress^a^ (MPa)	failure strain^a^ (%)	*T*_g,DMA_ ^b^ (°C)	*T*_NI,DMA_ ^b^ (°C)	*E*′_25°C_ ^b^ (MPa)	dissipation factor^b^ (°C)
TM_A,LCO_ 1.1/1	0.60 ± 0.09	(1.5 ± 0.2) × 10^–2^	489 ± 27	2	61	1.83	34
TM_A,LCO_ 1.25/1	1.9 ± 0.1	(2.6 ± 0.4) × 10^–1^	193 ± 17	10	75	5.45	34
TM_A,LCO_ 1.5/1	15 ± 2	(6.9 ± 0.8) × 10^–1^	170 ± 3	22	96	26.8	34
TM_SH,LCO_ 1/1.1	0.11 ± 0.02	(6.7 ± 0.3) × 10^–2^	2050 ± 140	–1	36	0.815	32
TM_SH,LCO_ 1/1.2	0.21 ± 0.02	(1.5 ± 0.8) × 10^–1^	711 ± 58	0	27	1.02	32
TM_SH,LCO_ 1/1.4	0.62 ± 0.07		372 ± 28	1	25	1.44	32
CT 1.1/1	22 ± 4	1.2 ± 0.1	119 ± 12	15	105	12.8	36
CT 1/1.5	0.052 ± 0.001	(5.5 ± 0.9) × 10^–3^	219 ± 8	2	53	0.717	47
CT 1/2	0.026 ± 0.002		297 ± 31	–5	35	0.472	36

a Determined by uniaxial tensile testing.

b Determined by DMA.

For LCEs prepared *via* chain extension, an increase in the DP_*n*_ of LCO precursors leads to higher deformability and reduced stiffness. This is because a higher DP_*n*_ results in a lower cross-linking density and longer chain segments between cross-linking points, allowing for higher chain mobility under applied stress. Consequently, a higher DP_*n*_ leads to higher failure strain and a lower Young modulus. More specifically, TM_SH_ networks exhibit higher failure strains and lower Young moduli than TM_A_ networks, with failure strains up to 2000% for TM_SH_ networks, compared to a maximum of 500% for TM_A_ networks. This difference is attributed to the lower cross-linking density in TM_SH_ networks, which enhances their deformability. In addition to Young modulus, threshold stress was also evaluated to determine the stress required to initiate mesogenic domain orientation and reach the soft elasticity plateau. As observed for Young modulus in TM_A_ and TM_SH_ networks, threshold stress increases with cross-linking density, since a higher network rigidity requires more stress to induce mesogenic chain segment orientation along the loading axis. Moreover, increasing the DP_*n*_ of LCO precursors extends the region of soft elasticity, allowing the material to withstand larger strain before stiffening. For instance, in TM_A_ 1.1/1, the soft elasticity region extends from 10% to 80% strain, while in TM_SH_ 1/1.1, it extends markedly, from 100% to 900% strain (Figure 5). In contrast, for TM_A_ 1.5/1, the soft elasticity region is much more restricted, extending only from 30% to 60% strain, and it is even missing for TM_SH_ 1/1.4. These networks with high cross-linking density have a behavior approaching LC networks. Overall, mechanical deformation of LCE samples prepared *via* chain extension is initially governed by mesogen reorientation, followed by the stretching of chain segments.

For LCEs prepared *via* chain transfer, increasing the EDDET molar content leads to higher flexibility and reduced stiffness. This is first due to the lower cross-linking density, caused by a higher number of chain transfer events, which explains the higher failure strains. Moreover, the presence of dangling thiols also induces a drastic softening of CT networks. For instance, CT 1/1.5 and CT 1/2 show lower Young moduli than all LCEs prepared *via* chain extension. This is consistent with literature reports indicating that an increase in the concentration of pendant chains decreases the elastic properties by reducing the fraction of elastically active chains in the network.^41^ Moreover, CT networks exhibit the narrowest range of failure strains, between 100% and 300%, which can be attributed to their network structure. Unlike LCEs formed *via* chain extension, CT networks are obtained through acrylate homopolymerization of RM257, leading to shorter segments between cross-linking points compared to the long oligomeric chains in TM_A_ and TM_SH_ networks. This results in reduced deformability. Additionally, as observed in LCEs prepared *via* chain extension, threshold stress values increase with cross-linking density. However, CT networks exhibit much more restricted soft elasticity regions, extending by only about 10% strain for all networks, even for CT 1/2. In CT networks, unlike in LCEs prepared *via* chain extension, all mesogens are directly attached to the network. As a result, a smaller deformation is required to induce mesogen alignment. Overall, during mechanical deformation of LCE samples prepared *via* chain transfer, mesogenic domain orientation occurs almost simultaneously with the stretching of chain segments. Understanding these differences in behavior under applied stress is important. LCEs prepared *via* chain extension are better suited for applications requiring high strain at low stress, while LCEs prepared *via* chain transfer offer higher stiffness.

To further investigate the influence of the synthesis route on the thermomechanical properties of LCEs under dynamic loading conditions, DMA was performed (Figure 5b). The transition temperatures, including *T*_g,DMA_ and *T*_NI,DSC_, along with *E*′_25°C_ and dissipation factors, are listed in Table 3. All LCEs show a broad tan δ peak between *T*_g,DMA_ and *T*_NI,DMA_ and a temporary drop in *E*′ at the nematic-to-isotropic transition. Overall, the trends observed for *T*_g,DMA_ and *T*_NI,DMA_ are similar to those obtained by DSC, though slight differences occur due to the differences between thermal and thermo-mechanical characterization techniques. Notably, for TM_SH_ networks, the trend in *T*_g,DMA_ values, taken at the peak of the tan δ curve, is more distinctly identified in DMA compared to DSC, providing a more accurate analysis of the transition. The storage modulus *E*′_25°C_ for each sample provides additional insight into the stiffness and elastic response of LCE networks under small oscillatory deformations at ambient temperature. The stiffness trends observed in Young modulus are similar to those in *E*′_25°C_, confirming that both parameters effectively capture the influence of cross-linking density and network structure of LCE networks, whether under static or dynamic loading. To assess the overall energy dissipation in the LC networks, the dissipation factor was quantified as the area under the tan δ curve between −40 °C and *T*_NI,DMA_. All the networks exhibit a dynamic soft elastic response, characterized by high tan δ values when heating above *T*_g,DMA_, followed by a return to minimum plateau value when *T*_NI,DMA_ is reached. No clear trend was observed in dissipation factor values which do not depend on cross-linking density and on the synthesis route.

Overall, the comparison between synthesis routes regarding the mechanical and thermo-mechanical properties of LCE networks allowed the identification of some of the same key structural factors as for the thermal properties. Cross-linking density is a key structural factor governing failure strain, Young modulus, *E*′_25°C_, and threshold stress. As cross-linking density decreases, LCEs exhibit greater failure strain, lower Young modulus and *E*′_25°C_, and reduced threshold stress. The distribution of mesogens within the network is a key structural factor governing the soft elasticity behavior. In chain extension, most mesogens are located within the polymer chains between cross-linking points, whereas in chain transfer, they are directly attached to the network, resulting in a more constrained soft elasticity region.

### Shape-Memory LCE Bilayer

After examining the differences in thermal, mechanical, and thermo-mechanical properties of the different LCE networks, their potential application in a shape-memory LCE bilayer was investigated. This shape-memory bilayer concept relies on combining two layers of LCE networks with distinct behaviors. In general, shape-memory polymers are programmed by deforming them into a temporary shape, which is then fixed by cooling them below a transition temperature such as *T*_g_ or melting temperature. When the shape memory is heated above this transition, increased chain mobility allows entropy-driven recovery of the original shape. For shape-memory LCEs, programming occurs within a specific temperature range, above *T*_g_ but below *T*_NI_, to allow strong mesogenic interactions. The material is first stretched to a defined strain at this specific temperature. It is then unloaded and allowed to stabilize at a temporary shape, demonstrating its fixity. It is finally heated to recover its initial shape. The most cross-linked samples from each synthesis route, TM_A_ 1.5/1, TM_SH_ 1/1.4, and CT 1.1/1, were excluded from the study due to their properties closely approaching those of LC networks. The fixity (*R*_f_), representing the shape retention of the networks, was assessed using DMA after applying 100% engineering strain and allowing 1 h of stabilization, all conducted at *T*_g,DMA_ + 10 °C. The fixity value was determined as the final strain plateau value after 1 h of stabilization.

The fixity curves are shown in Figure S4. *R*_f_ values were plotted as a function of *T*_NI,DMA_ to assess the suitability of these networks as potential layers in the shape-memory LCE bilayer actuator, as shown in Figure 6a. For all synthesis routes, *R*_f_ increased as cross-linking density decreased. This can be attributed to reduced constraints on mesogens, allowing them to better maintain their alignment when the material is unloaded. For the shape-memory LCE bilayer, TM_A_ 1.1/1 and TM_SH_ 1/1.2 were selected, as they exhibit the best *R*_f_ values at similar programming temperatures while showing different *T*_NI,DMA_, which is essential for achieving the best shape change. The shape recovery of TM_A_ 1.1/1 and TM_SH_ 1/1.2 was then assessed using DMA (Figure 6b). As in the fixity measurement, TM_A_ 1.1/1 and TM_SH_ 1/1.2 were stabilized at 12 and 10 °C, respectively. They were then stretched to 100% strain, unloaded, allowed to stabilize for 1 h, and subsequently heated at a rate of 10 °C/min. The unrecovered strain at maximum shape recovery for both networks was attributed to the DMA testing method. As expected, TM_SH_ 1/1.2 recovered at a lower temperature than TM_A_ 1.1/1. As observed in the derivatives of engineering strain as a function of temperature, TM_SH_ 1/1.2 completed its shape recovery between 10 and 70 °C, at which point TM_A_ 1.1/1 began its transition, occurring between 70 and 120 °C. A shape-memory LCE bilayer ∼1 mm thick was fabricated through two successive photo-cross-linking steps, with the top layer composed of TM_SH_ 1/1.2 and the bottom layer of TM_A_ 1.1/1. The bilayer underwent the same shape-memory cycle as the individual LCE networks. (1) It was first stabilized at 10 °C, corresponding to the programming temperature of TM_SH_ 1/1.2. (2) It was then stretched to 100% strain and left to stabilize in its temporary shape, where mesogens maintained their alignment. The transparency of the bilayer indicates that the bilayer is in a monodomain nematic state. When heated to room temperature, the bilayer underwent a self-wrapping deformation, rolling onto itself around an axis perpendicular to the mesogen alignment axis corresponding to the stretching direction. The rolling was directed toward the TM_SH_ 1/1.2 layer. As observed in the DMA curves, at 25 °C, TM_SH_ 1/1.2 had already begun its shape recovery. Consequently, during thermal actuation, the top layer contracted, while the bottom layer did not, leading to a rolling deformation. (3) When heated to 70 °C, the bilayer exhibited further rolling as TM_SH_ 1/1.2 completed its shape recovery, while TM_A_ 1.1/1 had not initiated its transition. (4) At 120 °C, the bilayer recovered its initial shape and became transparent, confirming its transition to the isotropic state. At this temperature, both networks had completed their shape recovery. When cooled, the bilayer kept its original shape and became white, indicating its transition to the nematic polydomain state. This cycle could be repeatedly triggered, demonstrating the LCE bilayer shape-memory behavior. Interestingly, this LCE bilayer can be tested in its polydomain state at room temperature without any previous stretching. When the bilayer is stretched at room temperature, where TM_SH_ 1/1.2 begins its shape recovery, an immediate rolling deformation occurs around this axis perpendicular to the stretching direction, as shown in Video S1. This behavior is particularly remarkable because it does not occur in a LCE film with the same thickness composed solely of either TM_A_ 1.1/1 or TM_SH_ 1/1.2 at room temperature, as shown in Video S2.

**Figure 6:**
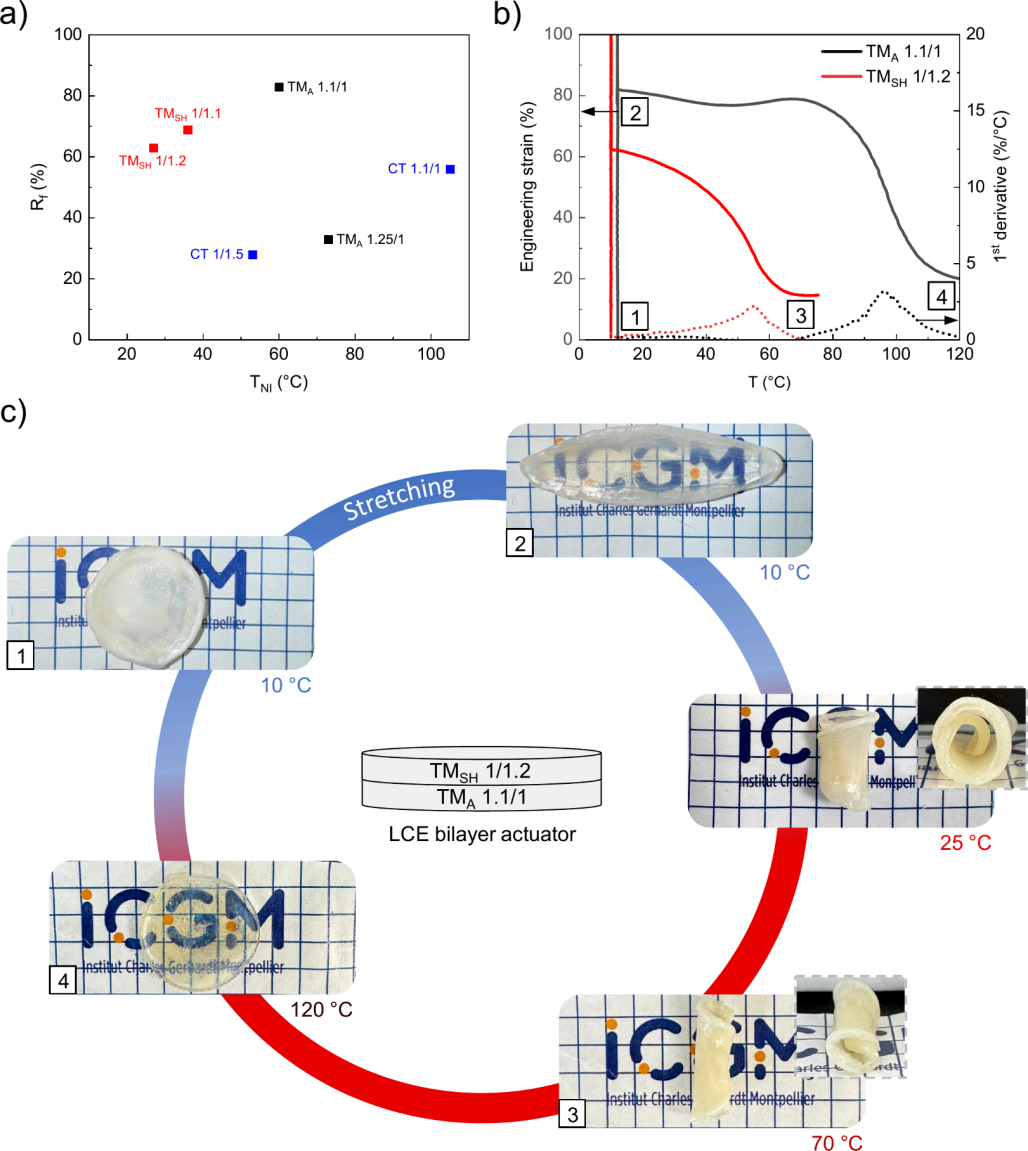
(a) Fixity (*R*_f_) of TM_A_ 1.1/1, TM_A_ 1.25/1, TM_SH_ 1/1.1, TM_SH_ 1/1.2, CT 1.1/1 and CT 1/1.5 as a function of their corresponding *T*_NI_. (b) Strain recovery behavior and the first derivative of engineering strain with respect to temperature for TM_A_ 1.1/1 and TM_SH_ 1/1.2. (c) Shape-memory behavior of the LCE bilayer with the top layer composed of TM_SH_ 1/1.2 and the bottom layer of TM_A_ 1.1/1. In all photos, each square in the background grid measures 0.5 cm. The use of the ICGM logo has been authorized by the institution.

## Conclusion

This study systematically investigates how different network synthesis strategies, chain extension and chain transfer, impact the properties of main-chain LCEs. Beyond establishing the range of properties achievable for each synthesis route, this study identifies key structural factors that are valid for both approaches. To establish these factors, the influence of critical parameters in each synthesis route is examined. For chain extension, we evaluated the effect of the chemical nature and length of LCOs, while for chain transfer, we assessed the impact of pendant thiol content and the occurrence of chain transfer events. For both synthesis routes, cross-linking density is a major key factor governing LCE properties. In chain extension, it is determined by the length of the LCOs, with longer oligomers resulting in lower cross-linking density. In chain transfer, it is determined by the content of EDDET, where a higher EDDET content increases the number of chain transfer events and the fraction of pendant thiols within the network, thereby reducing cross-linking density and the proportion of elastically active chains. For both approaches, as cross-linking density decreases, the resulting LCEs exhibit lower *T*_g_, higher Δ*H*_NI_, greater failure strain, lower Young modulus and *E*′_25°C_, reduced threshold stress, and enhanced fixity. The proportion of mesogenic units within the network is another key factor influencing LCE properties. In both chain extension and chain transfer, the LC content, dictated by the RM257/EDDET molar ratio, directly governs *T*_NI_. As the proportion of mesogenic units in the network increases, mesogenic interactions strengthen, requiring more thermal energy to disrupt the LC order and resulting in a higher *T*_NI_. Additionally, while TM_A_ networks exhibit relatively high *T*_NI_ values and TM_SH_ networks show particularly low *T*_NI_ values, CT networks provide the widest tunable range of *T*_NI_. The distribution of mesogens within the network, particularly whether cross-linking points are derived from mesogens, is also a major key factor governing LCE properties. In chain extension, most mesogens are located within the polymer chains between cross-linking points. In contrast, in chain transfer, all mesogens are directly attached to the network, as their terminal ends serve as potential cross-linking sites during acrylate homopolymerization. CT networks, with cross-linking points derived from mesogens, show noticeably more constrained soft elasticity regions and exhibit higher *T*_NI_ for similar LC content as mesogenic interactions are favored by cross-linking points. Then, we demonstrated the practical relevance of this study by fabricating a LCE bilayer with shape-memory behavior using TM_A_ 1.1/1 and TM_SH_ 1/1.2 networks. This bilayer exploits the distinct thermomechanical responses of the two networks, as they exhibit the best fixity values at similar programming temperatures while showing different *T*_NI_. As a result, the bilayer undergoes a controlled rolling deformation during shape recovery, driven by the contrasting phase transition temperatures of both layers.

Overall, this study provides guidelines for tailoring LCE properties simply by selecting the appropriate synthesis route. By identifying the key structural factors that govern LCE behavior in chain extension and chain transfer strategies, it establishes a deeper understanding of structure–property relationships in LCEs. Furthermore, it shows that by using one type of mesogen and one type of dithiol, it is possible to obtain very different properties simply by changing the synthesis conditions. These insights pave the way for the development of new synthesis approaches, expanding the design possibilities for next-generation LCE networks with enhanced properties.

## Supplementary Material







## Notes

^1^H NMR spectra, Raman spectra, and fixity measurement curves (PDF)
Video S1 shows when the bilayer is stretched at room temperature, where TM_SH_ 1/1.2 begins its shape recovery, an immediate rolling deformation occurs around this axis perpendicular to the stretching direction (AVI)
The behavior seen in Video S1 is particularly remarkable because it does not occur in a LCE film with the same thickness composed solely of either TM_A_ 1.1/1 or TM_SH_ 1/1.2 at room temperature, as seen this Video S2 (AVI)
