# Supplementary material for: Influence of Network Synthesis Strategies on Liquid Crystal Elastomer Properties
Source: Macromolecules. 2025 Jul 25;58(15):7823–36. doi: 10.1021/acs.macromol.5c01037 (PMC12451743; doi:10.1021/acs.macromol.5c01037)
Supplement: Supplementary file 1 [file ma5c01037_si_001.pdf]

Supporting information for

Influence of Network Synthesis Strategies on Liquid Crystal Elastomer Properties

Rakine Mouhoubi[a], Jason Richard[b], Vincent Lapinte[a], Sébastien Blanquer[a]*

[a]Institut Charles Gerhardt Montpellier (ICGM), CNRS, Université de Montpellier, ENSCM, 34293 Montpellier, France

[b]Institut Européen des Membranes (IEM), CNRS, Université de Montpellier, ENSCM, 34090 Montpellier, France

E-mail: sebastien.blanquer@umontpellier.fr

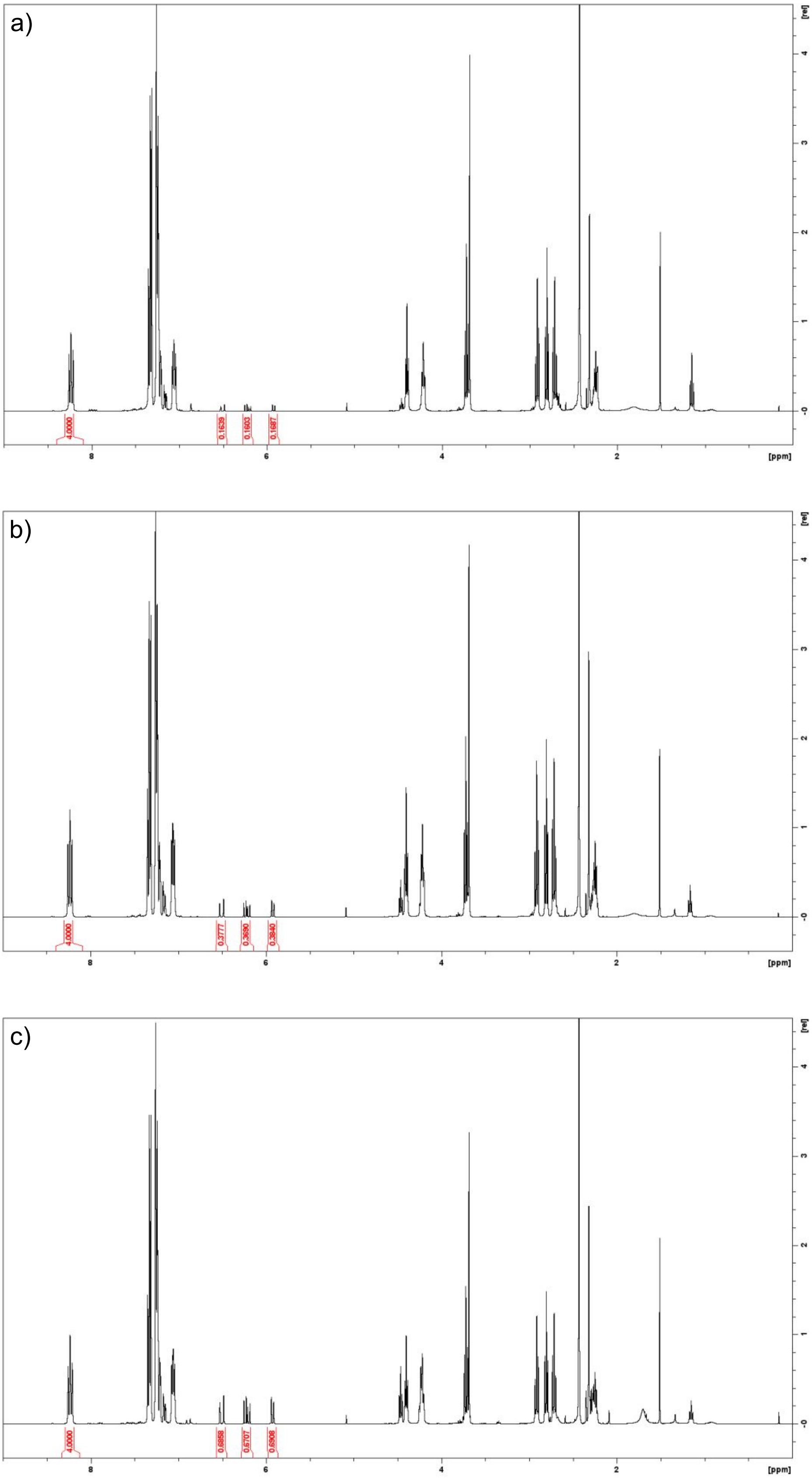

Figure S1: ^{1}H NMR spectra of (a) $TM_{A,\ LCO}$ 1.1/1, (b) $TM_{A,\ LCO}$ 1.25/1, and (c) $TM_{A,\ LCO}$ 1.5/1.

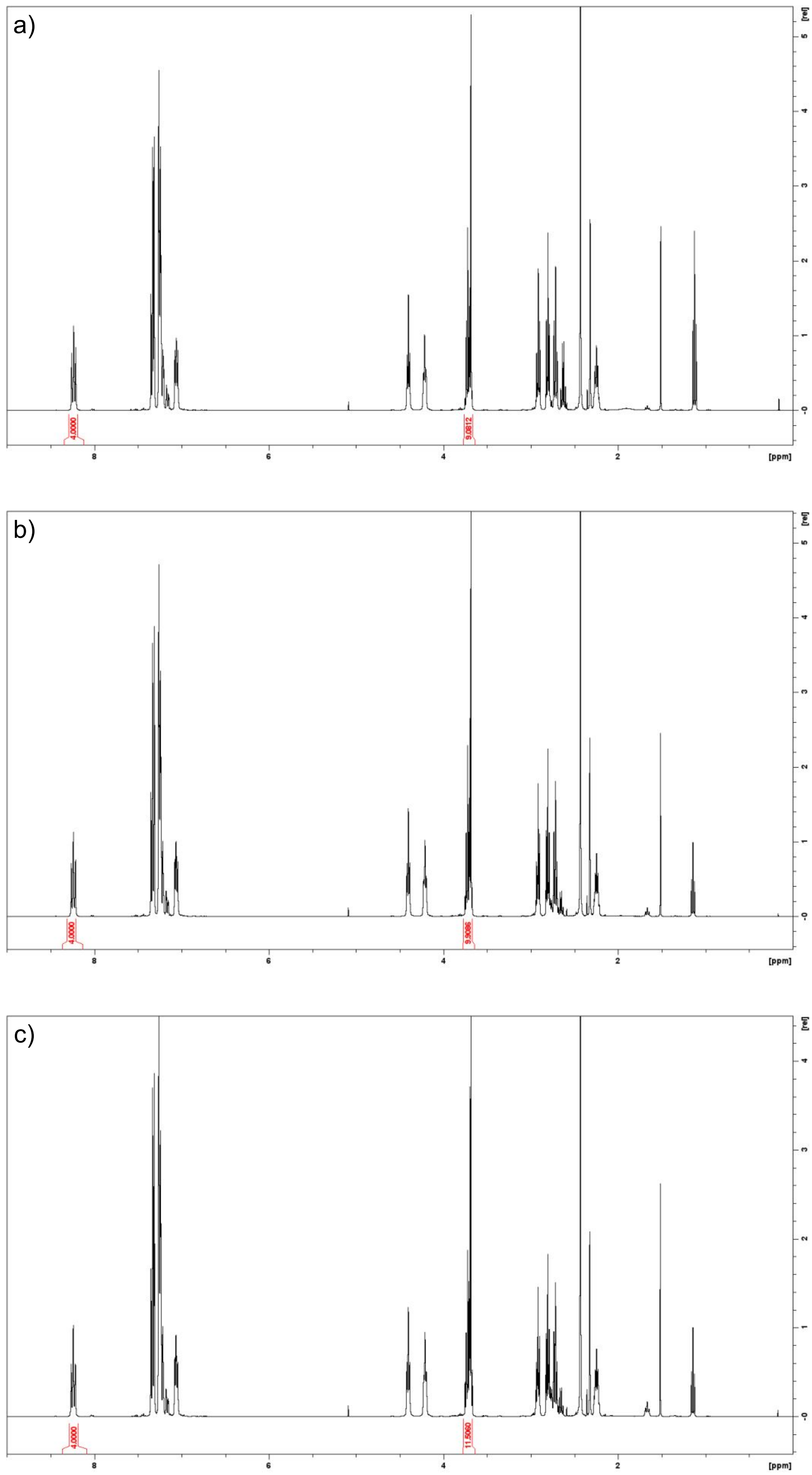

Figure S2: ^{1}H NMR spectra of (a) $TM_{A,\ SH}$ 1/1.1, (b) $TM_{SH,\ LCO}$ 1/1.2, and (c) $TM_{SH,\ LCO}$ 1/1.4.

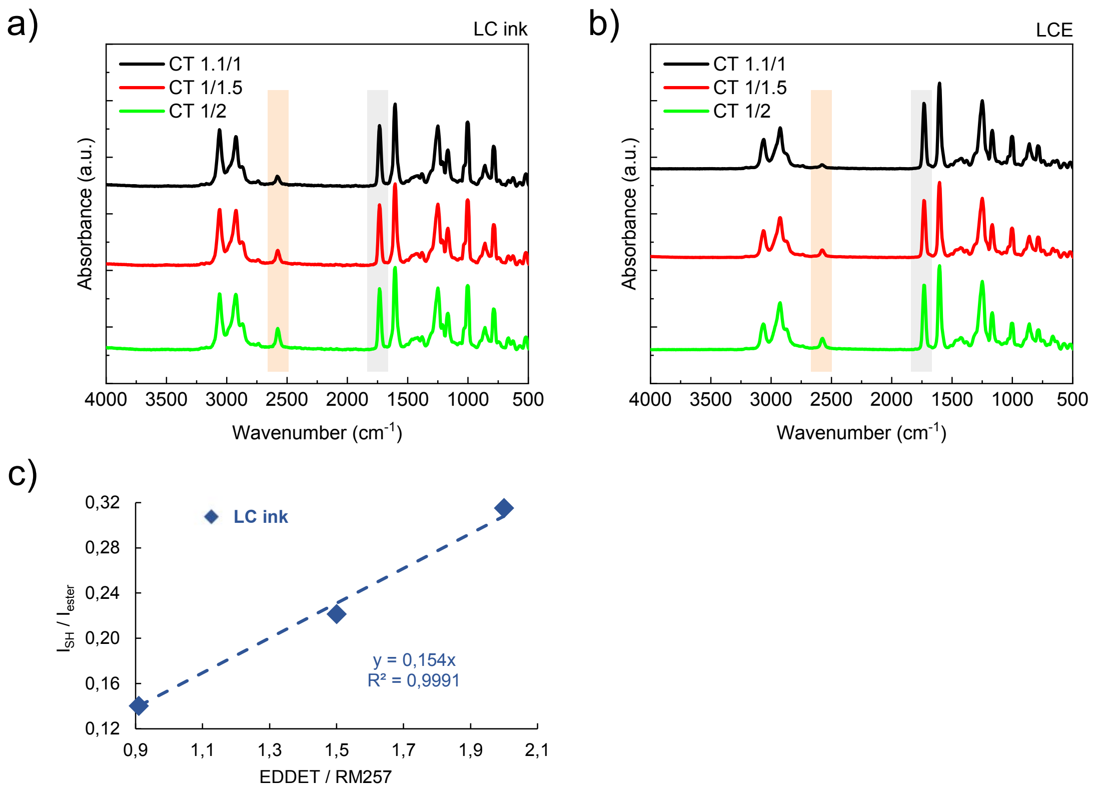

Figure S3: Normalized Raman spectra of (a) LC inks, and (b) CT networks, with highlighted S-H and ester peaks at 2577 cm^{-1} and 1730 cm^{-1}, respectively. (c) I_{SH}/I_{ester} intensity ratio as a function of EDDET/RM257 ratio.

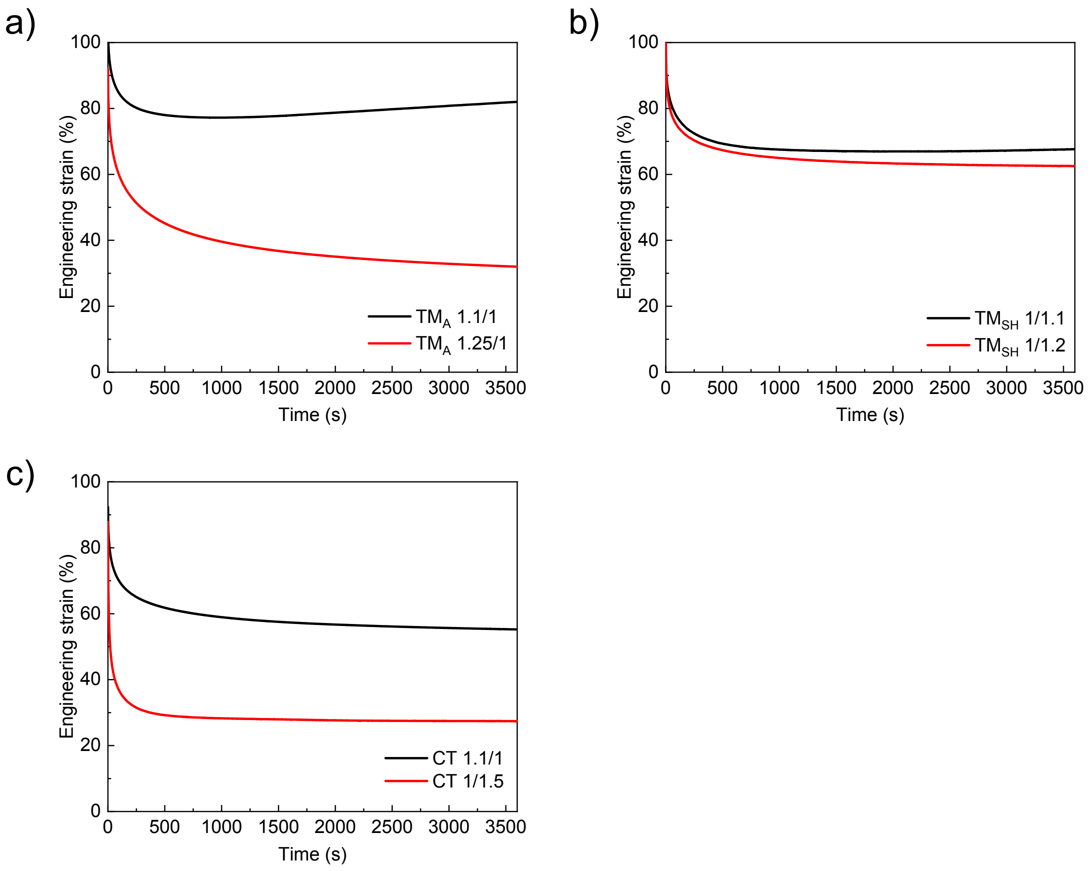

Figure S4: Fixity measurement curves for TM_A 1.1/1, TM_A 1.25/1, TM_{SH} 1/1.1, TM_{SH} 1/1.2, CT 1.1/1, and CT 1/1.5.
